# Socioeconomic status and adolescent cannabis use: a Swedish cohort study

**DOI:** 10.1186/s42238-025-00334-3

**Published:** 2025-09-23

**Authors:** Patrik Karlsson, Mats Ekendahl, Isabella Gripe, Jonas Raninen

**Affiliations:** 1 Department of Social Work, Stockholm University, Stockholm, SE-106 91 Sweden; 2 The Swedish Council for Information on Alcohol and Other Drugs (CAN), Stockholm, Sweden; 3 Department of Clinical Neuroscience, Karolinska Institutet, Stockholm, Sweden; 4 Centre for Alcohol Policy Research, La Trobe University, Melbourne, Australia

**Keywords:** Cannabis, Adolescents, Socioeconomic status, Parental education, Cohort study

## Abstract

**Background:**

The evidence is mixed regarding how socioeconomic status (SES) it is related to cannabis use among adolescents. This study assessed the association between parental SES, measured as the highest level of completed education, and past 12 month cannabis use in older adolescents.

**Method:**

Self-reported survey data from the first and second wave of a nationwide cohort study (Futura01) were used (*n* = 3328). Register information on parental education was linked to the survey data. Two measures of cannabis use were considered: any use during the past 12 months, and use 10 + times during the past 12 months. Control variables included demographics, family and school variables, conduct and emotional problems, and cannabis use at baseline. Multilevel Poisson regression was used to assess the associations.

**Results:**

Adolescents having parents with low SES had a lower risk for any cannabis use during the past 12 months, ranging from RR = 0.71 (95% CI = 0.49–1.01) in the unadjusted model to RR = 0.61 (95% CI = 0.42–0.87) in the most adjusted model compared to adolescents with parents having high SES. Compared to those with parents with high SES, those with parents with intermediate SES had lower risk for any cannabis use past 12 months, with RRs ranging from 0.79 (95% CI = 0.59–1.07) in the unadjusted model to RR = 0.71 (95% CI = 0.53–0.95) in the fully adjusted model. For use 10 + times, non-significant associations were observed.

**Conclusion:**

Adolescents with parents with lower SES had a lower risk of any past 12 months cannabis use. For more frequent use, no statistically significant associations were observed.

## Introduction

It is widely known that socioeconomic status (SES) predicts health-related outcomes, and that SES in fact may be a “fundamental cause of disease” (Link and Phelan 1995). Lower SES is associated with a range of health behaviours, including for example tobacco use and physical inactivity (Pampel et al. 2010), and these patterns have been identified among adolescents as well (Hansen & Chen, 2007). The link between SES and adolescent cannabis use, however, is complex, and the literature is far from consistent.

SES is a multidimensional concept, but generally refers to people’s position in the social stratification system of society, and is typically assessed by measures such as education, occupation or income (Adler and Ostrove 1999; Pampel et al. 2010). For adolescents, SES is usually defined at the parental or family level. However, very few studies on parental SES and adolescent cannabis use have used objective measures, instead relying on adolescents’ reports on their parents’ education (Gerra et al. 2020; Gripe et al. 2021; Legleye et al. 2012) or other self-defined information pertaining to SES (Gerra et al. 2020; Lowtian et al., 2021). This implies a potential and important source of bias, misclassification, which in turn may lead to erroneous estimates of the link (e.g., Höfler 2005).

Available research across different countries presents a complicated picture of how SES is associated with cannabis use during adolescence or young adulthood. Adolescents with parents with higher education have been found to be more (Charitonidi et al. 2016; Gripe et al. 2021; Humensky 2010; Legley et al., 2012; Patrick et al. 2012) or less (Aschengrau et al. 2021) likely to have used cannabis, whereas other studies find no significant association (Gerra et al. 2020; Gripe et al. 2024; see also Hansen & Chen, 2007). The reasons for these conflicting findings are difficult to pinpoint, but may be due to both actual differences across contexts and to measurement and other methodological issues. The association between SES and substance use in adolescence may also vary at different ages, potentially because of how normative use is (Van Ryzin et al. 2012), and different patterns have been observed for different indicators of SES (e.g., parental vs. own educational level, Charitonidi et al. 2016). Research focusing on more frequent use has shown that adolescents having parents with lower education are more likely to have used cannabis frequently (Gripe et al. 2021; Legley et al., 2012), whereas other studies only confirm this pattern for some other adolescent reported SES measures (Gerra et al. 2020).

The lack of consistent findings impedes theoretical developments regarding how SES may shape cannabis and other substance use among adolescents. Different mechanisms are likely to be at work if higher SES would be related to more prevalent cannabis use compared to if the opposite would be the case. Here it is worth emphasizing that higher SES groups may be more advantaged on well-known risk factors for cannabis use, including truancy, conduct problems and low parental monitoring (cf. Henry et al. 2009; Van Ryzin et al. 2012; Karlsson et al. 2018&^2024^). There is thus a risk that SES and these other risk factors, if not considered, cancel each other out, and that the association between SES and cannabis use becomes underestimated.

This study assesses the association between adolescent SES – measured as the highest level of parental education – and cannabis use in a nationwide sample of older adolescents in Sweden, focusing both on unadjusted and adjusted associations. It adds to prior work by using an objective measure of parental education, derived from national registers in Sweden, and by using longitudinal cohort data. A range of models pertaining to the association are presented, considering both any use during the past 12 months and more frequent use. Variables adjusted for include sex, parental country of birth, family structure, parental monitoring and support, truancy, conduct and emotional problems, as well as prior cannabis use. As the literature is mixed, it is difficult to formulate specific hypotheses regarding the direction and magnitude of the association. However, given the considerations above, in the case of a higher unadjusted risk of cannabis use for lower SES groups, we expect the controlled associations to be closer to the null. Conversely, to the extent that the unadjusted relationship is essentially zero, or if the risk of cannabis use is higher in higher SES groups, we expect the adjusted associations to be more strongly positive than the unadjusted one.

## Methods

### Sample

The data used for the study are derived from a Swedish longitudinal cohort study (Futura01) of Swedish adolescents who attended 9th grade in compulsory school during 2017 (i.e., when most were 15 or 16 years old). Besides the baseline data (t1) we also use data from the first follow up in 2019 (t2) when most participants attended second grade in upper secondary school (i.e., when most were 17 or 18 years old).

At t1, a sample of 500 Swedish schools were drawn by Statistics Sweden, using a probability-proportional-to size approach. Of the schools invited, 343 agreed to participate, corresponding to a response rate of 68.6%. Participating schools did not differ significantly from non-participating schools regarding students’ average grades, immigrant background or parental education (Raninen 2020). At each school, one class was randomly selected. Students who were present in school during the day of data collection filled out a questionnaire during school-hours (paper-and-pen), and they also provided their social security numbers, which then made it possible to collect register data (see more below). In all, 5541 students consented to participate, provided readable and correct social security numbers, and did provide reasonable responses on key variables. This yielded a participation rate at the student level of 82% (Raninen 2020).

At t2, postal letters were sent to participants inviting them to again take part in the study. Participants could either fill in a paper-and-pen questionnaire or a web-based one. Most participants choose the latter option (83%). A total of 3999 eligible individuals took part in t2, corresponding to a retention rate of 72%. There was differential attrition between t1 and t2, where for example females, adolescents with higher SES, with at least one parent born in Sweden, who lived with both their parents, and who had not tried cannabis at t1 were significantly more likely to participate in t2.

This study included those participants who took part in t2 and who had non-missing data on all variables included here (*n* = 3328, or 83%). The largest share of missing values was found for cannabis use at baseline (*n* = 297, 8.0%), followed by parental SES (*n* = 160, 4.0%). There were no statistically significant differences in any of the cannabis use measures, sex, truancy, and emotional problems between participants with non-missing data on all included variables and those with missing data. However, included participants had significantly higher levels of SES, a larger share had at least one parent born in Sweden, more lived with both parents, and they scored more favourably on parental monitoring and support, as well as on conduct problems.

### Variables

#### Outcome variables

Two measures of *cannabis* use at t2 were used: any use during the past 12 months, and use 10 + times during the past 12 months. Participants who reported that they had used illicit drugs during the past 12 months were asked how many times they had used various substances during this time frame, ranging from 0 to 40 times or more. Regarding the first measure, individuals who had used marijuana and/or hashish at least 1 or 2 times were coded as 1, and those who had not used any of these substances the past 12 months were coded as 0.

To compute the measure of 10 + times use during the past 12 months, we combined the responses to both the marijuana and the hashish question and then coded those whose combined use corresponded to use 10 + times as 1 and those whose combined use was lower as 0. Although it would have been preferable to also include a measure of more frequent use, data consideration did not allow for this (due to sparse cells). However, it may be noted that among Swedish adolescents in this age who have ever used an illicit drug, such as cannabis (about 15%), 70% have used at the most 2–6 times the past 12 months (CAN, 2024). Thus, in this context, the 10 + times measure captures a rather rare phenomenon.

#### Exposure

The primary exposure variable is *SES*, measured as parental education as of 2017 (t1). Information on mother’s and father’s highest level of completed education was collected from Statistic Sweden’s Longitudinal Integrated Database for Health and Labour Market Studies. This information was obtained by first linking participating students’ social security numbers with their parents’ social security numbers through Statistic Sweden’s Multigenerational register. In the register, the highest completed level of education is registered according to seven different values, with higher values corresponding to higher education.

To obtain a manageable measure of parental SES, mothers’ and fathers’ education were first recoded into two separate variables with three values each: (1) a maximum of two years of upper secondary education, (2) three years of upper secondary education, and (3) tertiary education. This approach to categorising parental education has been used in prior Swedish research (e.g., Låftman and Östberg 2024). Each participant was then assigned the highest level of parental education in their household, considering whom they lived with. Participants living with both their parents were assigned the highest value of these, and the same was true for those living separately with both their mother and their father. Participants living with only one of their parents were assigned this parent’s highest level of education. Individuals who did not live with any of their parents were excluded (*n* = 69).

#### Control variables

All control variables were measured at t1. Information on *foreign background* was collected from Statistic Sweden’s Population Register (RTB) database and also concerned parents. We distinguished between three groups: (1) at least one parent born in Sweden, (2) both parents born in Europe (including the Nordics but not Sweden), and (3) both parents born outside of Europe. Information on participants’ *sex* was derived from their social security number. *Family structure* was based on a question asking participants with whom they live, and were coded as 1 (both parents) and 0 (other arrangements).

*Parental monitoring* and *parental support* were both measured with two separate variables that were added together and reverse coded, with higher scores indicating higher parental monitoring and higher parental support, respectively. The study also included variables related to *conduct problems* (CP), *emotional problems* (EP), *truancy*, and *cannabis use at t1*. Both CP and EP were measured by the Strengths and Difficulties Questionnaire (SDQ, Goodman 1997) where each scale is measured with five items. The values of the respective items were added together (after reverse coding one item for the CP scale), yielding a scale ranging from 0 to 10. Cronbach alpha was 0.51 for CP in the analytical sample, and 0.70 for EP. While the Cronbach’s alpha for CP was lower than satisfactory, prior work on the same material indicates that the scale is unidimensional (Karlsson et al. 2024). *Truancy* measured whether students skipped school at least once a month the last semester. Finally, we included an indicator of whether participants had ever used cannabis at t1, distinguishing between 1 (yes) and 0 (no).

### Statistical analyses

Students with different SES were first compared on all other variables. These comparisons entailed χ^2^ -test for categorical variables and *F*-test for continuous ones.

Multilevel, modified Poisson regressions (Zou 2004) were run to estimate the associations (relative risks, RR, with accompanying 95% confidence intervals) between SES and the two cannabis use measures, adjusting for the other variables. The modified Poisson regression includes robust standard errors in order not to give inflated *p*-values (Zou 2004) and here it was run within a multilevel framework. As students were nested in school classes at t1, the regressions included a random school class intercept. Totally six models were estimated for each outcome, beginning with a crude model and ending with a model adjusting for all the control variables. The first adjusted model (model 2) adjusted for sex and parental country of birth, and the second (model 3) also adjusted for family structure, as well as for parental monitoring and parental support. Model 4 added truancy, and model 5 also adjusted for conduct and emotional problems. The fully adjusted model (model 6) also included life time use of cannabis at t1. Associations at *p* ≤ 0.05 were considered statistically significant.

As a robustness test, given that 17% of the sample had missing data on at least one variable, we also run the statistical analyses on multiple imputed data (not shown in the results section below). As the data included both continuous and categorical variables, Multiple Imputation by Chained Equations (MICE, see White et al. 2011) was used. We imputed two separate sets of 20 data sets each, one for any past 12 months use and all the predictor variables, and one with use 10 + times and all the predictor variables. Some of the binary outcomes were perfectly predicted in the imputation steps, i.e., some combinations of outcome and predictor variables included empty cells (White et al. 2010). To handle this, augmented regression was used, which briefly entails adding a small set of additional observations to the data so that such perfect prediction is avoided (White et al. 2010). Poisson regressions with cluster robust standard errors were then used in the analysis step. While these results were overall similar to the main analyses, the differences between SES groups in any past 12 months use were significant in all models, including the unadjusted one.

The study was not pre-registered and the findings should be considered exploratory. All analyses were conducted in Stata 17.0 (StataCorp 2021).

## Results

### Descriptive statistics

Descriptive statistics are presented in Table 1. About 9% had used cannabis during the past 12 months, and somewhat more than 3% had used 10 + times. Any past 12 month cannabis use was significantly higher in the highest SES group (*p* = 0.036). Cannabis 10 + times use was most common in the lowest SES group but these differences were not significant. The highest SES group was by far the largest one (63.37%), and the lowest SES group was the smallest (16.20%). Overall, there were some central differences between SES groups. In the lowest SES group, there was a smaller share with at least one parent born in Sweden and the share living with both parents was also smallest in this group. The level of parental monitoring and support were higher in the highest SES group compared to the two other groups, but the magnitude of the differences was small. Truancy was least common in the highest SES group. The highest SES group had the lowest level of CP, although the differences were relatively minor. No statistically significant differences across SES groups were observed for **sex**, EP or cannabis use at t1.

**Table 1:** Descriptive statistics for included variables (*n* = 3328), overall and by socioeconomic status (SES)

	All		Lowest SES		Intermediate SES		Highest SES		
	*N*	% / M (SD)	*N*	% / M (SD)	*N*	% / M (SD)	*N*	% / M (SD)	*p*-value^a^
**Follow-up**									
Cannabis LYP 1 + times	298	8.95	37	6.86	52	7.65	209	9.91	0.036
Cannabis LYP 10 + times	109	3.28	19	3.53	18	2.65	72	3.41	0.582
**Baseline**									
SES									
Lowest	539	16.20							
Intermediate	680	20.43							
Highest	2109	63.37							
Parental country of birth									
At least 1 born in Sweden	2835	85.19	375	69.57	600	88.24	1860	88.19	< 0.001
At least 1 born in Europe	164	4.93	53	9.83	23	3.38	88	4.17	
Both born outside of Europe	329	9.89	111	20.59	57	8.38	161	7.63	
Lives with both parents	2398	72.06	313	58.07	457	67.21	1628	77.19	< 0.001
Boy	1492	44.83	234	43.41	299	43.97	959	45.47	0.609
Parental monitoring (range 2–10)	3328	8.93 (1.56)	539	8.79 (1.73)	680	8.86 (1.60)	2109	8.99 (1.51)	0.013
Parental support (range 2–10)	3328	8.90 (1.75)	539	8.70 (1.89)	680	8.81 (1.83)	2109	8.98 (1.68)	0.001
Monthly truancy	334	10.04	80	14.84	77	11.32	177	8.39	< 0.001
CP (range 0–10)	3328	1.67 (1.52)	539	1.98 (1.67)	680	1.89 (1.54)	2109	1.52 (1.45)	< 0.001
EP (range 0–10)	3328	3.44 (2.35)	539	3.48 (2.49)	680	3.63 (2.34)	2109	3.38 (2.31)	0.050
Cannabis LTP	166	4.99	32	5.94	31	4.56	103	4.88	0.513

SES = socioeconomic status; CP = conduct problems; EP = emotional problems; LYP = last year prevalence; LTP = lifetime prevalence; Lowest SES = 2 years upper secondary education or less; Intermediate SES = 3 years upper secondary; Highest SES = tertiary education

^a^Chi2-test for categorial variables and f-test (one-way ANOVA) for continuous variables

### Regression models

Table 2 presents results from the regression models predicting any past 12 month use of cannabis, at t2, from SES and the other variables measured at t1. In the unadjusted model, the highest SES group had the highest risk for cannabis use but none of the comparisons were statistically significant. When adjusting for the other variables in model 2 to 5, all but one of the comparisons between the highest SES group and the other two SES groups were significant, and the associations also became stronger compared to the unadjusted model. Compared to the highest SES group, the lowest SES group had a lower risk of cannabis use that ranged from 33% lower in model 2 to 41% lower in model 5. The differences were somewhat smaller between the highest and the intermediate group. In the significant models, the risk of cannabis use in the intermediate SES group ranged from 24% lower in model 3 to 32% lower in model 5, compared to the highest SES group. As to the other variables, being a boy and having less parental monitoring and support as well as playing truant were significantly associated with cannabis use. Not living with both parents were significantly related to cannabis use in controlled models but not when also adjusting for prior cannabis use. Having at least one parent born in Sweden (compared to having both parents born outside of Europe) was significantly associated with cannabis use in adjusted models 4 and 5 when not when adjusting for cannabis use at t1. Baseline use of cannabis was strongly related to past 12 months use at t2, with a more than three times higher risk (RR = 3.15).

**Table 2:** Modified multilevel Poisson regression of any cannabis use during the past 12 months. Relative risks (RR) and 95% confidence intervals

	Model 1	Model 2	Model 3	Model 4	Model 5	Model 6
	RR [95% CI]	RR [95% CI]	RR [95% CI]	RR [95% CI]	RR [95% CI]	RR [95% CI]
**Any use past 12 months**						
SES^a^						
Lowest	0.71	0.67*	0.63*	0.61**	0.59**	0.61**
	[0.49 1.01]	[0.46 0.97]	[0.43 0.92}	[0.42 0.87]	[0.41 0.84]	[0.42 0.87]
Intermediate	0.79	0.76	0.73*	0.70*	0.68*	0.71*
	[0.59 1.07]	[0.57 1.03]	[0.54 0.98]	[0.52 0.95]	[0.51 0.92]	[0.53 0.95]
Highest	1	1	1	1	1	1
Parental country of birth						
At least 1 born in Sweden		1	1	1	1	1
At least 1 born in Europe		1.01	0.97	0.94	0.93	0.99
		[0.62 1.64]	[0.58 1.60]	[0.57 1.55]	[0.56 1.54]	[0.61 1.61]
Both born outside of Europe		0.79	0.66	0.64*	0.64*	0.65
		[0.50 1.23]	[0.42 1.04]	[0.41 0.98]	[0.42 0.99]	[0.42 1.01]
Boy		1.85***	1.73***	1.77***	1.85***	1.82***
		[1.46 2.33]	[1.38 2.18]	[1.41 2.23]	[1.46 2.33]	[1.44 2.30]
Lives with both parents		0.65***	0.70**	0.74*	0.76*	0.80
		[0.51 0.83]	[0.55 0.88]	[0.59 0.94]	[0.60 0.96]	[0.63 1.03]
Parental monitoring			0.85***	0.87***	0.89***	0.91***
			[0.81 0.89]	[0.83 0.92]	[0.84 0.94]	[0.86 0.96]
Parental support			0.90***	0.91***	0.93**	0.94*
			[0.85 0.95]	[0.86 0.96]	[0.88 0.98]	[0.89 0.99]
Monthly truancy				2.05***	1.86***	1.49**
				[1.57 2.68]	[1.41 2.45]	[1.13 1.96]
CP					1.10***	1.05
					[1.04 1.17]	[0.99 1.11]
EP					1.03	1.05
					[0.98 1.08]	[1.00 1.10]
Cannabis LTP						3.15***
						[2.32 4.27]
*N*	3328	3328	3328	3328	3328	3328

^a^ Lowest = 2 years upper secondary education or less; Intermediate = 3 years upper secondary; Highest = tertiary education

RR = relative risk; CI = confidence interval; SES = socioeconomic status; CP = conduct problems; EP = emotional problems; LTP = lifetime prevalence

***p ≤ 0.001; **p ≤ 0.01; *p ≤ 0.05

Table 3 shows the results from the regression models predicting 10 + times use of cannabis during the past 12 months. The relative risks for SES were relatively similar to those presented in Table 2 but none were statistically significant. However, this being the case, the magnitude of the RRs changed as more other variables were adjusted for. As to the other variables, boys had a substantially higher risk than girls for 10 + times cannabis use, with a more than three-fold higher risk in the fully adjusted model (RR = 3.07). Truancy was also strongly associated with use 10 + times in model 4 and 5 but this association was largely reduced when adjusting for baseline cannabis use. Living with both parents was also clearly associated with the outcome, with the risk being about 0.5 in the fully adjusted model (RR = 0.52). Higher levels of parental monitoring and support were inversely related to the risk of 10 + times use of cannabis at t2 throughout models. Baseline use of cannabis had a strong link to cannabis use 10 + times (RR = 6.58). 

**Table 3:** Modified multilevel Poisson regression of cannabis use 10 or more times during the past 12 months. Relative risks (RR) and 95% confidence intervals

	Model 1	Model 2	Model 3	Model 4	Model 5	Model 6
	RR [95% CI]	RR [95% CI]	RR [95% CI]	RR [95% CI]	RR [95% CI]	RR [95% CI]
**10 + times use past 12 months**						
SES^a^						
Lowest	1.04	0.87	0.79	0.75	0.72	0.76
	[0.64 1.69]	[0.52 1.45]	[0.47 1.32]	[0.45 1.22]	[0.44 1.18]	[0.46 1.28]
Intermediate	0.79	0.72	0.67	0.62	0.60	0.67
	[0.48 1.29]	[0.44 1.18]	[0.40 1.10]	[0.37 1.04]	[0.36 1.01]	[0.41 1.10]
Highest	1	1	1	1	1	1
Parental country of birth						
At least 1 born in Sweden		1	1	1	1	1
At least 1 born in Europe		1.41	1.32	1.29	1.30	1.49
		[0.67 2.94]	[0.60 2.89]	[0.59 2.78]	[0.60 2.79]	[0.70 3.14]
Both born outside of Europe		0.63	0.48	0.46	0.47	0.48
		[0.28 1.45]	[0.20 1.15]	[0.20 1.07]	[0.20 1.09]	[0.21 1.10]
Boy		3.16***	3.00***	3.11***	3.17***	3.07***
		[2.12 4.70]	[2.03 4.42]	[2.11 4.60]	[2.07 4.87]	[1.98 4.77]
Lives with both parents		0.38***	0.41***	0.45***	0.46***	0.52**
		[0.25 0.56]	[0.28 0.61]	[0.31 0.66]	[0.31 0.68]	[0.35 0.78]
Parental monitoring			0.82***	0.85***	0.86**	0.90*
			[0.75 0.90]	[0.77 0.93]	[0.78 0.95]	[0.82 0.99]
Parental support			0.85***	0.86***	0.87***	0.90*
			[0.78 0.92]	[0.79 0.94]	[0.80 0.94]	[0.83 0.98]
Monthly truancy				2.68***	2.44***	1.57*
				[1.74 4.12]	[1.58 3.76]	[1.01 2.44]
CP					1.09*	0.99
					[1.00 1.19]	[0.90 1.07]
EP					1.02	1.06
					[0.94 1.12]	[0.97 1.15]
Cannabis LTP						6.58***
						[4.01 10.78]
*N*	3328	3328	3328	3328	3328	3328

^a^ Lowest = 2 years upper secondary education or less; Intermediate = 3 years upper secondary; Highest = tertiary education

RR = relative risk; CI = confidence interval; SES = socioeconomic status; CP = conduct problems; EP = emotional problems; LTP = lifetime prevalence

***p ≤ 0.001; **p ≤ 0.01; *p ≤ 0.05

## Discussion

Against the background of conflicting findings in this area (Aschengrau et al. 2021; Charitonidi et al. 2016; Gerra et al. 2020; Gripe et al. 2021&^2024^; Hansen & Chen, 2007; Humensky 2010; Legley et al., 2012; Lowtian et al., 2021; Patrick et al. 2012), this study explored how socioeconomic status (SES) is related to cannabis use among a nationally representative sample of older Swedish adolescents. Using objective, register-based data on parental education as the measure of SES, a higher risk of any past 12 months use of cannabis at age 17/18 among those with higher SES was observed in most of the adjusted models. For cannabis use 10 times or more during the same time frame, similar patterns were found but the associations were not statistically significant.

The study supports our expectation that the association between SES and cannabis use, to the extent that it is actually positive, will be underestimated if not adjusting for baseline differences between SES groups that in turn are associated with cannabis use. In fact, the relative risk of cannabis use between the highest and the other SES group was smallest in the unadjusted regression analyses, and it was not significant for any of the outcomes. The descriptive analyses also showed that the highest SES group was more advantaged on several other variables (e.g., truancy, conduct problems) that are known to be associated with cannabis use (e.g., Henry et al. 2009; Karlsson et al., 2018; 2024; Van Ryzin et al. 2012). As a consequence, it seems imperative to consider other risk factors for cannabis use that are less prevalent in the highest SES group when assessing the association. If not, the contribution of SES and the other risk factors may cancel each other out, which in turn may indicate that SES is unrelated to cannabis use.

The analyses used an objective measure of parental education. This contrasts with most prior work where parents’ education or other indicators of SES are measured by adolescent self-reports (Gerra et al. 2020; Gripe et al. 2021; Legleye et al. 2012; Lowtian et al., 2021). Relying on such measures theoretically increases the risk of misclassification (cf. Höfler 2005), which may impact the estimates. However, a Swedish cross-sectional study (Gripe et al. 2021) observed a slightly higher risk of life-time cannabis use at age 17/18 (RR = 1.17 in the most adjusted model) for those adolescents who self-reported that at least one parent had tertiary education. It is therefore conceivable that the potential problems with adolescent-reported parental education are modest. Nonetheless, it should be noted that in the Gripe et al. (2021) study, adolescents were 17/18 years of age when they reported their parents’ education, and the potential problem with misclassification may be bigger in studies that use adolescent reports at earlier ages. Also, a key-benefit of using objective, register measures is that there will be fewer missing values on parental SES, which are often quite high in studies using self-reports.

The study illustrates that the risk of any use of cannabis during the past 12 months is higher among young Swedes with high compared to low parental SES, net of risk factors such as truancy and parental monitoring. Still, cannabis use is rather uncommon among Swedish adolescents (CAN, 2023) and the absolute risks for cannabis use were small across SES groups. This points towards caution when considering implications of the findings for prevention initiatives, not least given that our definition of “frequent use” is inclusive, and that sporadic use of cannabis is less likely to lead to severe consequences compared to heavier use (cf. Hall 2015). While this study has identified a social gradient in adolescent cannabis use, future research should consider more severe cannabis-related outcomes, preferably using objective measures of SES. Research on the mechanisms underlying the gradient is also needed. As of now we can only speculate why Swedish adolescents with higher SES appear more prone to experiment with cannabis. Future work may consider factors such as financial resources, perceived risk and parental attitudes towards cannabis use.

### Strengths and limitations

Some limitations should be mentioned. While parental SES (and country of birth) were measured using register data, data on the other variables were derived from self-reports with all their known limitations. Cannabis, and other substance use, is prone to be underreported, meaning that the prevalence figures in this study are probably underestimated. Differential levels of underreporting among different SES groups are also plausible, which would introduce further problems. However, although recent estimates suggest cannabis use to be underreported among young Swedes, no clear differences between different SES groups in underreporting were observed (Andersson et al. 2023). Thus, there are no obvious reasons for assuming that different SES groups differ in how likely they are to disclose information about cannabis use. Nonetheless, such potential differences may exist for the other variables included. It should also be noted that adolescents with extensive social and individual problems are probably underrepresented in the sample, not least at t2, and that there were systematic differences in attrition from t1 to t2 across groups. For example, females and those with a higher parental SES were significantly more likely to participate at t2. This, however, does not necessarily affect the associations observed in a substantial way (see Canivet et al. 2021). It should also be noted that we only used one type of objective SES measure and that other measures such as parental occupation or parental income may capture additional, important facets of parental SES. Future studies, including those based on the Futura01 data, should explore how other, objective SES measures relate to cannabis use among adolescents.

Key-strengths of the study includes nationwide cohort data with a relatively high retention rate (72%) and the objective measure of SES. Another strength is the large sample size, which allowed us to assess the association between SES and cannabis use while simultaneously adjusting for a number of control variables. Although several variables were based on self-reports, most of them were based on measures widely used in the literature.

## Conclusion

Using an objective measure of parental SES, the study shows that older Swedish adolescents with parents with lower SES are less likely to have used cannabis any time during the past 12 months. For more frequent use, no statistically significant associations were observed. In order not to underestimate the positive SES gradient in adolescent cannabis use, it is crucial to control for important baseline differences in known risk factors for cannabis use among different SES groups.

## Data Availability

The data that support the findings of this study are managed by Karolinska Institutet. The Futura01 survey include sensitive information on individuals, as defined by the Swedish Personal Data Act (Personuppgiftslagen, PUL, SFS 1998:204; § 13). Restrictions apply to the availability of these data, which were used under license for this study. Data are available from jonas.raninen@ki.se given approval from the Swedish Ethical Review Authority. Access to the data for the purpose of the current study was based on being a member of a research team with ethical approval for analyses of data from Futura01. Researchers who qualify by getting ethical approval from Swedish authorities can request access to data from the data holder.
